# *Enterobacter roggenkampii* ST422 with highly conjugative IncP plasmid carrying *bla*_KPC-2_ gene from Lebanon: a case report

**DOI:** 10.1128/asmcr.00239-25

**Published:** 2026-03-31

**Authors:** Souad Fayad, Dina Daaboul, Delphine Girlich, Monzer Hamze, Hassan Mallat, Laurent Dortet, Fouad Dabboussi, Issmat I. Kassem, Bogdan I. Iorga, Saoussen Oueslati, Marwan Osman, Thierry Naas

**Affiliations:** 1 Laboratoire Microbiologie Santé et Environnement (LMSE), Doctoral School of Sciences and Technology, Faculty of Public Health, Lebanese University 63572, Tripoli, Lebanon; 2 Team "Resist" UMR1184, "Immunology of Viral, Auto-Immune, Hematological and Bacterial diseases (IMVA-HB", INSERM, Université Paris-Saclay, CEA, IO Healthy, Faculty of Medicine 27048, Le Kremlin-Bicêtre, France; 3 Department of Biology, Faculty of Arts and Sciences, Holy Spirit University of Kaslik 67028, Jounieh, Lebanon; 4 Clinical Laboratory, Nini Hospital, Microbiology Department 237177, Tripoli, Lebanon; 5 Bacteriology Ward, University Hospital Bicêtre, APHP, Le Kremlin-Bicêtre, France; 6 French National Reference Center for Carbapenemase producing Enterobacterales, Hôpital Bicêtre, APHP, Le Kremlin-Bicêtre, France; 7 Center for Food Safety and Department of Food Science and Technology, University of Georgia 92569, Griffin, Georgia, USA; 8 Faculty of Agricultural and Food Sciences, American University of Beirut 11238, Beirut, Lebanon; 9 Université Paris-Saclay, CNRS, Institut de Chimie des Substances Naturelles 57474, Gif-sur-Yvette, France; 10 Department of Neurosurgery, Yale University School of Medicine 537605, New Haven, Connecticut, USA; 11 Yale Institute for Global Health 198927 , New Haven, Connecticut, USA; Pattern Bioscience, Austin, Texas, USA

**Keywords:** antimicrobial resistance, carbapenemase, KPC-2, *Enterobacter roggenkampii*, One Health, Lebanon, case report

## Abstract

**Background:**

The *Enterobacter cloacae* complex (ECC) includes opportunistic pathogens that can be carbapenem resistant, thus complicating treatment regimens. Here, we characterized a carbapenem- and colistin-resistant ECC O89H7 isolate expressing KPC carbapenemase.

**Case Summary:**

ECC O89H7 was isolated in January 2021 from an axillary swab performed during routine screening for multidrug-resistant (MDR) bacteria from a patient after 39 days of hospitalization in the intensive care unit (ICU) for severe COVID-19 pneumonia at Nini hospital (Tripoli, Lebanon). The ECC O89H7 was identified as *E. roggenkampii* (*Er*) belonging to sequence type (ST)422 and contained eight different plasmids as revealed by whole-genome sequencing (WGS). *Er* O89H7 was predicted to be a human pathogen (96.3%), harboring 51 virulence factors as well as genes conferring resistance to heavy metals and quaternary ammonium compounds. *Er* O89H7 was highly drug resistant, including resistance to carbapenems and colistin. The resistome revealed six β-lactamase genes: the chromosome-encoded *bla*_MIR-3_ cephalosporinase, *bla*_LAP-2_ and *bla*_SHV-12_ encoded on a 115-kb IncM-1 plasmid, and *bla*_OXA-10_*, bla*_TEM-40_, and *bla*_KPC-2_ on a mobilizable IncP-6 plasmid of 51 kb, as revealed by mating-out assays.

**Conclusion:**

Here, we have characterized a human pathogenic *Er* ST422 harboring *bla*_KPC-2_ carbapenemase on an IncP-6 plasmid from Lebanon. *Er* O89H7 represents a major health threat due to limited therapeutic options, especially because novel β-lactam/inhibitor combinations are not available in Lebanon. Our results highlight an urgent need for improved carbapenemase screening and detection capacity in clinical laboratories and for enhanced genomic surveillance of MDR bacteria to implement intervention strategies to control their spread in Lebanon and beyond.

## INTRODUCTION

The *Enterobacter cloacae* complex (ECC) comprises a group of closely related bacterial species that are increasingly recognized as important opportunistic pathogens (1). These species, frequently associated with antimicrobial resistance (AMR), are isolated from a wide range of sources across the human-environment continuum, highlighting their ecological adaptability and potential role in the dissemination of AMR determinants (2–4). Among ECC members, *Enterobacter roggenkampii* (*Er*) has recently drawn attention due to the identification of carbapenem-resistant isolates recovered from patients and environmental reservoirs in healthcare settings (5, 6). Unlike dominant ECC species such as *E. hormaechei*, it shows high colistin resistance but lower virulence (serum susceptibility) and ceftazidime resistance (7).

Although increasingly described worldwide, species-level identification of *Er* has not been reported in Lebanon, likely due to limitations of standard microbiological methods in differentiating it from other ECC members and possible gaps in local surveillance (1, 8, 9). To address these epidemiological gaps, national screening initiatives aimed at detecting and reporting multidrug-resistant (MDR) pathogens, including carbapenemase-producing *Enterobacterales* (CPE), in both hospital and community settings have been conducted (1, 3, 10–12). Our previous work has revealed a predominance of OXA-48-like variants in Lebanon, with a progressive shift toward NDM-5-producing *Enterobacterales* in recent years, which has been observed in both clinical and community isolates, including those retrieved from animals (3, 11, 13). This shift, coupled with the increasing prevalence of carbapenem resistance, highlights the circulation of AMR determinants in both healthcare and community reservoirs. In contrast, although prevalent worldwide, *bla*_KPC_ gene variants have been rarely reported in Lebanon and across the broader Middle East and North Africa (MENA) region (3, 13–15).

Here, we report an *Er* O89H7 isolate from Lebanon co-harboring six β-lactamase genes, including *bla*_KPC-2_ carbapenemase. This report extends the known geographic distribution of this species and offers novel insights into its resistance determinants within the MENA region.

## CASE PRESENTATION

A carbapenem-resistant ECC O89H7 was isolated in 2021 from an axillary swab performed during routine culture-based screening of MDR bacteria on non-selective MacConkey agar followed by antimicrobial susceptibility testing (AST) of recovered gram-negative colonies. The isolate originated from an intensive care unit (ICU) patient hospitalized with severe COVID-19 pneumonia after 39 days of hospitalization at a major healthcare facility that provides medical services to an estimated 1 million people across North Lebanon (16). The patient had no history of travel and was repeatedly negative for carbapenem-resistant Gram-negatives during initial screenings, suggesting acquisition during hospitalization. Isolate O89H7 was initially identified using matrix-assisted laser desorption/ionization–time of flight–mass spectrometry (MALDI-TOF-MS) (Bruker Daltonics, Bremen, Germany; Biotyper score > 2.3) as ECC and later confirmed as *Er* using average nucleotide identity score (ANI, calculated using pyANI [v0.2.12]) that was highest (98.52) with *Er* CP017184 strain, the microbial classification engine Centrifuge and *hsp60* sequence. Phylogeny resulted in close clustering with an *Er* CP017184 strain previously isolated from a clinical sample in Germany (data not shown) (17–19).

Antimicrobial susceptibility of antibiotics available in Lebanese hospitals was assessed by disc diffusion antibiogram as part of clinical care and by broth microdilution in the research laboratory (Sensititre, ThermoFisher, Grenoble, France) and interpreted according to European Committee on Antimicrobial Susceptibility Testing (EUCAST; https://www.eucast.org) 2025 guidelines except for apramycin, neomycin, and chloramphenicol, which were interpreted using veterinarian breakpoints set by CA-SFM vétérinaire 2023 (https://www.sfm-microbiologie.org/wp-content/uploads/2023/06/CASFM_VET2023.pdf) and Clinical and Laboratory Standards Institute (CLSI VET01S ED6:2024; https://clsi.org/shop/standards/vet01/). These antibiotics were tested for resistance profiling and epidemiological insight, rather than to guide treatment. *Escherichia coli* clinical breakpoints were used for nitrofurantoin and fosfomycin, as no breakpoints for ECC exist for these antimicrobials. The isolate exhibited resistance to 23 of 37 tested antimicrobials spanning more than three clinically relevant antimicrobial classes, consistent with an MDR phenotype as defined by Magiorakos et al. (20) (Table 1). The isolate was susceptible only to newly released β-lactam antibiotics, including meropenem-vaborbactam, ceftazidime-avibactam, aztreonam-avibactam, and cefiderocol, but these antibiotics are not available for patient treatment in Lebanese hospitals. The isolate is resistant to carbapenems and colistin, both considered last resort drugs in Lebanon, and to newly released β-lactam antibiotics, including imipenem-relebactam, leaving treatment options limited to alternative agents such as trimethoprim-sulfamethoxazole, fosfomycin, and selected aminoglycosides, which significantly complicates clinical management. The laboratory-developed Carba NP Test, a colorimetric biochemical assay detecting carbapenemase activity (21), detected significant carbapenem-hydrolyzing activity; NG-Test CARBA 5 lateral flow immunoassays (NG-Biotech, Guipry, France) identified KPC (3, 22, 23).

**TABLE 1:** Antimicrobial susceptibility patterns of *E. roggenkampii* O89H7 isolated at Nini Hospital, Tripoli, Lebanon, and of its transconjugant *E. coli* J53 harboring pErO89H7_5 plasmid (O123D5)

Antibiotic class	Antibiotic name	MIC (µg/ml) for:	
		*E. roggenkampii* O89H7	*E. coli* J53(pErO89H7_5)^*a*^
β-lactams	Mecillinam	**>16**	**16**
	Cefotaxime	**>8**	**8**
	Ceftazidime	**>32**	**32**
	Cephalexin	**>128**	**128**
	Aztreonam	**>16**	16
	Cefepime	**16**	**16**
	Ceftolozane-tazobactam	**>16**	**16**
	Cefoxitin	**32**	**32**
	Ertapenem	**>8**	**8**
	Imipenem	**32**	**16**
	Meropenem	**16**	**16**
	Ceftazidime-avibactam	0.25	1
	Aztreonam-avibactam	0.5	0.5
	Cefiderocol	0.25	1
	Imipenem-relebactam	2	2
	Meropenem-vaborbactam	0.25	0.25
Fluoroquinolones	Norfloxacin	**>4**	**>4**
	Levofloxacin	**4**	**4**
	Ciprofloxacin	**2**	**2**
	Delafloxacin	**>2**	**0.5**
Aminoglycosides	Gentamicin	≤1	≤1
	Tobramycin	≤1	≤1
	Amikacin	≤4	≤4
	Apramycin	1	≤0.5
	Neomycin	≤4	≤4
	Streptomycin	**32**	**32**
Cyclins	Tetracycline	**8**	**16**
	Tigecycline	**2**	**1**
	Eravacycline	**1**	**1**
Others	Chloramphenicol	**>16**	**16**
	Florfenicol	**16**	**16**
	Colistin	**>8**	≤0.25
	Nitrofurantoin	64 (I)	≤32
	Fosfomycin	≤4	≤4
	Trimethoprim	**>8**	**8**
	Sulfamethoxazole	≤1	≤1
	Sulfamethoxazole and trimethoprim	≤32	≤32

^*a*^ Broth microdilution susceptibility testing results were interpreted using the European Committee on Antimicrobial Susceptibility Testing (EUCAST; https://www.eucast.org) 2025 guidelines, except for apramycin, neomycin and chloramphenicol, which were interpreted using veterinarian breakpoints set by CA-SFM vétérinaire 2023 (https://www.sfm-microbiologie.org/wp-content/uploads/2023/06/CASFM_VET2023.pdf). For tigecycline and eravacyline, *E. coli* breakpoints of ≤0.5 were used. MIC values considered resistant are shown in bold.

**Fig 1:**
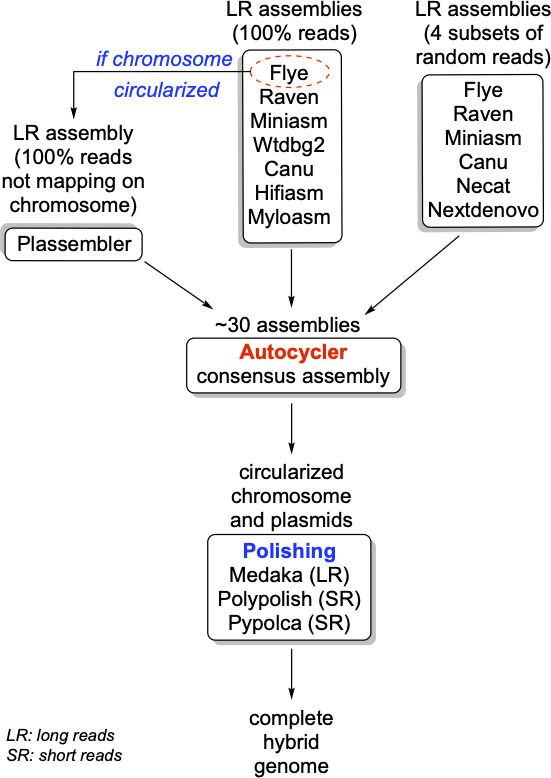
Genome assembly and bioinformatic pipeline. The Nanopore raw reads were first assembled with six different softwares: Flye version 2.9.4 (26), Miniasm version 0.3 (27), and Raven version 1.8.3 (28), all three as implemented in Dragonfly version 1.2.1 (https://github.com/rpetit3/dragonflye), Canu version 2.2 (29), Wtdbg2 version 2.5 (30), Hifiasm version 0.25.0 (31), Myloasm version 0.2.0 (32), and Plassembler version 1.8.0 (33). The whole raw read data set was then subsampled in four subsets using the protocol implemented in Autocycler (34). Each of these subsamples was assembled with six softwares (Flye, Miniasm, Raven, Canu, Necat [31], and NextDenovo [35]) using the scripts included in the Autocycler distribution. The resulting ~30 assemblies were used with an Autocycler to generate a consensus assembly, with the chromosome and the plasmids fully circularized. The genome was polished sequentially with Medaka version 2.1.1 (https://github.com/nanoporetech/medaka), Polypolish version 0.6.1 (36), and Pypolca version 0.4.0 (37), then further rotated using Dnaapler (38) to facilitate the comparison of contigs. For comparison, the genome was also assembled with SKESA version 2.5.1 (16) starting from the Illumina raw short reads. The quality of the assembly was evaluated using SQUAT (39), and the contig circularization was assessed from the Autocycler output. The multilocus sequence typing (MLST) was performed using MLST version 2.23.0 (https://github.com/tseemann/mlst), and the gene annotation was performed with PROKKA version 1.14.5 (40). Antimicrobial resistance motifs and plasmid incompatibility groups were determined using Abricate version 1.0.1 (https://github.com/tseemann/abricate) with the Resfinder, NCBI AMRFinder, CARD, and PlasmidFinder databases (25).

**Fig 2:**
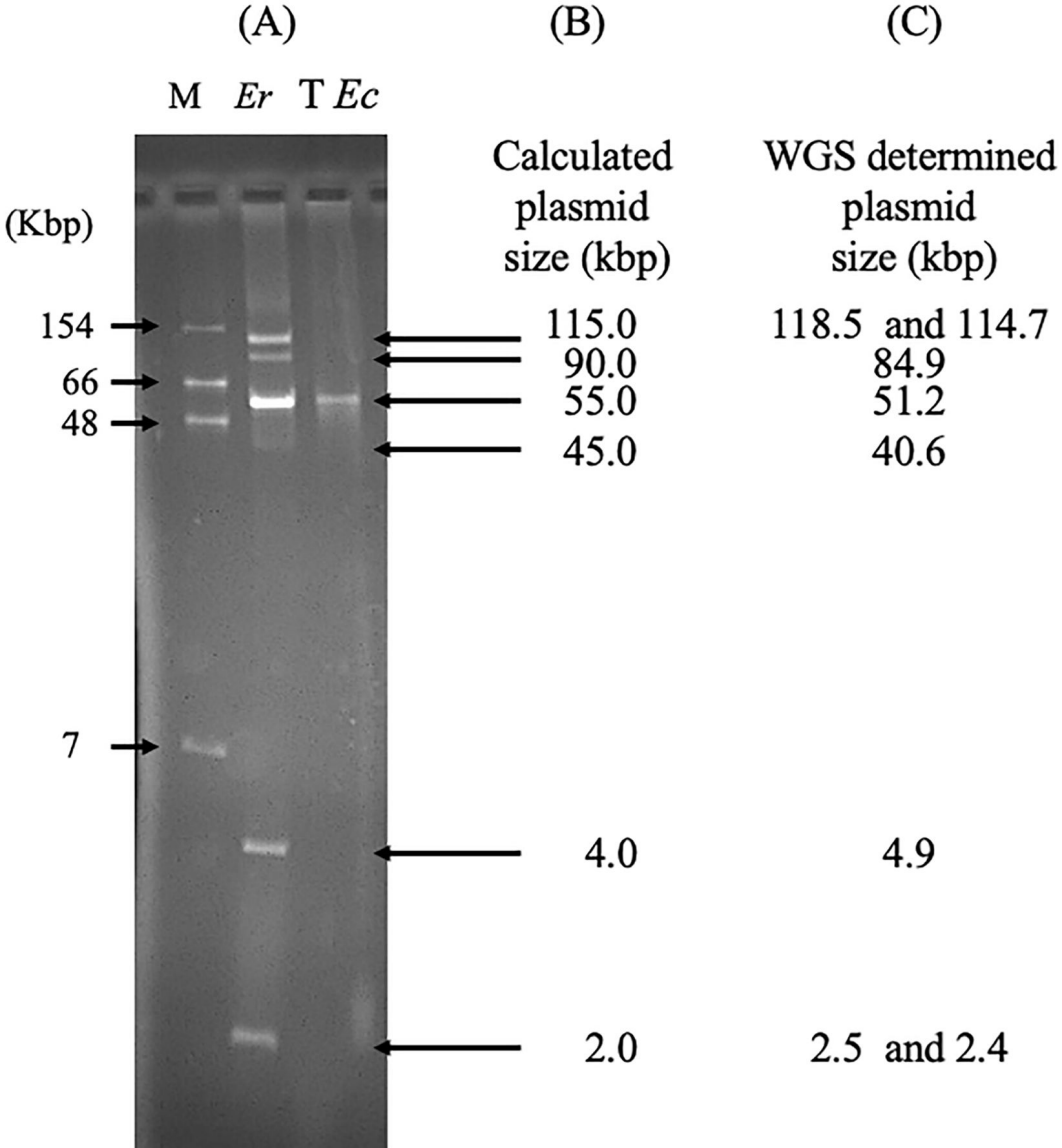
Analysis of Kieser extracted plasmids. (**A**) Agarose (0.7%) gel electrophoresis of extracted plasmids of *E. roggenkampii* O89H7 (lane *Er*) and its KPC positive *E. coli* J53 O123D5 transconjugant (lane T *Ec*) as previously described (12). Lane M corresponds to *E. coli* NCTC 50192, harboring four plasmids of known size: 7, 48, 66, and 154 kb (12). (**B**) Calculated band sizes based on migration distances using those of the size marker. (**C**) Plasmid sizes present in *Er* O89H7 based on WGS results.

Short read sequencing on a NextSeq 500 sequencer (Illumina, San Diego, CA, USA) and long-read sequencing on MinION (Oxford Nanopore, Oxford, UK) were performed as previously described (24). The bioinformatic pipeline used to assemble the reads is detailed in Fig. 1. The assembly of the genome of *Er* O89H7 revealed nine circularized contigs. The first one corresponded to a chromosome of 4,831,396 bp in size with a GC content of 55.41%. The other eight contigs corresponded to circularized plasmids belonging to different incompatibility groups as revealed with PlasmidFinder (25): plasmids pErO89H7_2 (118,514 bp; IncFIB/IncFII), pErO89H7_3 (114,730 bp; IncM1), pErO89H7_4 (84,963 bp; unknown Inc), pErO89H7_5 (51,233 bp; IncP6), pErO89H7_6 (40,615 bp; unknown Inc), pErO89H7_7 (4,958 bp; Col440II), pErO89H7_8 (2,519 bp; ColIMGS31), and pErO89H7_9 (2,429 bp; unknown Inc). Electrophoresis of Kieser extracted plasmids of *Er*O89H7 revealed the presence of six bands corresponding to at least six plasmids of ca. 2, 4, 45, 55, 90, and 115 kb in size based on gel migration (Fig. 2). The plasmid pairs pErO89H7_8 (2.5 kb) and pErO89H7_9 (2.4 kb) and pErO89H7_2 (118.5 kb) and pErO89H7_3 (114.7 kb) are too similar in size to be resolved on a 0.7% agarose gel and therefore migrate as single bands.

PathogenFinder v2 and VirulenceFinder v2.0.5 predicted Er O89H7 to be a likely human pathogen (96.3% probability) carrying 51 virulence factors (Table 2) (41, 42). Whole-genome sequencing (WGS) identified blaKPC-2 along with five additional β-lactamase genes: the chromosomal *bla*_MIR-3_ cephalosporinase, plasmid-encoded ESBL *bla*_SHV-12_, and narrow-spectrum β-lactamases *bla*_OXA-10_*, bla*_LAP-2_, and a truncated *bla*_TEM-40_ gene. In total, the resistome comprised eight plasmid-borne AMR genes across multiple antimicrobial classes (Table 2). The isolate belonged to ST422 (43), a rare sequence type reported only once in the PubMLST database and not previously described in the literature.

**TABLE 2:** Genome analyses of *Enterobacter roggenkampii* O89H7

Antimicrobial family	Resistance gene(s) (% identity)	Genomic location
ꞵ-lactams	*bla*_MIR-3_ (100.00%)^*a*^	Chromosomal (intrinsic gene)^*b*^
	*bla*_KPC-2_ (100.00%), *bla*_OXA-10_ (100.00%), *bla*_TEM-40_ (93.51%)	pErO89H7_5 (IncP-6)
	*bla*_LAP-2_ (100.00%), *bla*_SHV-12_ (100.00%)	pErO89H7_2 (IncM1)
Macrolides	*mph(A)* (99.57%)	pErO89H7_5 (IncP-6)
Amphenicols	*cmlA1* (99.92%)	pErO89H7_5 (IncP-6)
Fluoroquinolones	*qnrVC4* (100.00%)	pErO89H7_5 (IncP-6)
	*qnrS1* (100.00%)	pErO89H7_2 (IncM1)
	GyrA (S83L), ParC (S57T, S129A), ParE (I355T)	Chromosomal acquired mutations
Trimethoprim	*dfrA14* (99.59%)	pErO89H7_5 (IncP-6)
Aminoglycosides	*ant(3″')-Ia_1* (99.75%)	pErO89H7_5 (IncP-6)
	*aac(6′)-Ib* (99.46%), *aadA1* (99.75%)	Chromosomal (intrinsic gene)
Chloramphenicol, ciprofloxacin, nalidixic acid, trimethoprim	*OqxB* (89.49%), OqxA (86.48%)	Chromosomal (intrinsic gene)
Colistin^*c*^	MgrB (Insertion of VW at positions 1 and 2, M3S, K4E, V12I, A42P) PhoP (F141L, N145D, N165S, H217Q) PhoQ (L4I, M5L, M9L, N67D, Q69R, S71T, V102I, L133I, K141Q, P168L, D169N, V178I, R190M, G193S, V196I, M298L, G464S) PmrA (A19G, A71I, D72N, C143Q, S145D, L146Q, P147A, R218S) PmrB (K90N, Q91K, Y110F, H132S, Q135K, A163S, S172A, A173T, I184V, D209E, T213E, E218G, A221T, P233A, A260S, T269P, A270R, Q271S, T275R, V227L, S278A, V279L, I280S, D281I, Q282R, G283V, P284L, G285E, I286L, D287M, E288K, A289R, H290T, R291D, Q292S, S293Q, I294L, T295P, E296S, R297A, R298V, D302T, Q303S, R304A, Y305T, G307A, S308V, L310W, G311A, L312A), frameshift at position 312 resulting in a premature stop codon	Chromosomal acquired mutations
Virulence genes^*d*^		
*acrA, acrB, cheB, cheD, cheR, cheW, cheY, cheZ, chuA, csgD, entA, entB, fcl, fepB, fepC, fepD, fepG, flgG, flgH, flgI, flhA, flhC, flhD, fliA, fliG, fliI, fliM, fliN, fliP, fliQ, galU, gmd, gmhA, lpcA, htpB, kdsA, lpxC, luxS, manB, motA, ompA, ppdD, rcsB, rfaD, rfaE*		Chromosomal (intrinsic gene)

^*a*^ Acquired antimicrobial drug resistance genes detected by ResFinder v4.1 using 99% identity as cut-off and ≥50% coverage to ensure full-length gene detection.

^*b*^ Plasmid replicons detected by Plasmidfinder v2.1 (25).

^*c*^ Amino acid changes in proteins involved in colistin resistance were analyzed using the genome of the ECC 43444 as the reference sequence (accession number NZ_JZYC01000000).

^*d*^ Virulence genes were screened against Virulence Factor Database (VFDB) using 70% identity and 60% coverage, due to the lack of *Enterobacter*-specific genes in VFDB. Candidates were verified with RAST annotations and BLASTx, and only those confirmed by RAST or showing 100% identity and coverage in BLASTx were retained (41).

**Fig 3:**
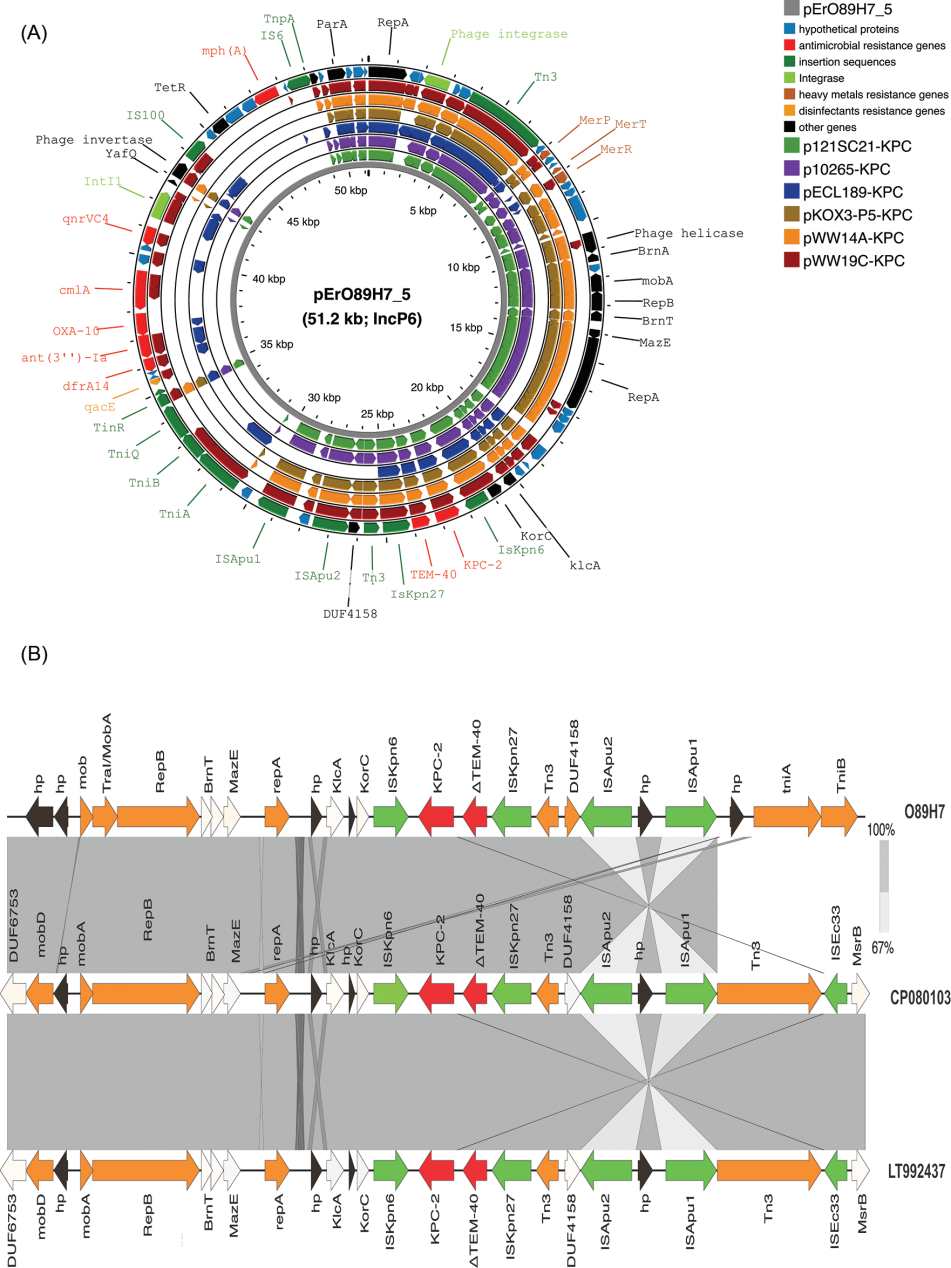
Plasmid analysis of a *bla*_KPC-2_-harboring *Enterobacter roggenkampii* O89H7 strain. (**A**) Plasmid comparisons of the 51,233-bp pErO89H7_5 IncP-6 plasmid generated using Proksee. From inside to outside: the first circle represents scale (in kbp); the second circle represents pErO89H7_5 (our isolate); the third through eighth circles represent plasmid similarity with six reference plasmids (with each color corresponding to a different reference plasmid). Colored regions in the concentric circles indicate ≥95% nucleotide identity with the reference plasmids, and white regions indicate no similarity. The outer circle shows the position of antimicrobial resistance genes (in red), insertion sequences (dark green), integrase (light green), disinfectant resistance genes (quaternary ammonium resistance genes: orange), heavy metal resistance genes (mercury resistance genes: brown), other genes (in black), and unknown open reading frames (hypothetical proteins: blue). (**B**) Linear comparison of ~14 kb containing *bla*_KPC-2_ gene of plasmid pErO89H7_5 of *E. roggenkampii* O89H7 isolate, pWW14A-KPC2 from *Klebsiella quasipneumoniae* strain WW14A (accession number CP080103), and p121SC21-KPC2 from *Citrobacter freundii* (accession number LT992437). Dark gray regions denote shared regions of homology (>95% nucleotide similarity). Genes are represented by arrows and colored according to functional classification: insertion sequences (IS) in green, β-lactamase genes in red, hypothetical proteins in black, and all other genes in white. The IS*Kpn6–bla*_KPC-2_*–*IS*Kpn27* cassette is conserved in similar IncP6 plasmids (also shown in panel A) across various bacterial species. The following genes are indicated: *hp* (hypothetical protein), *mob* (mobilization relaxase), *traI* (conjugative transfer protein), *repB* (plasmid replication initiation protein B), *brnT* (toxin, BrnTA system), *mazE* (antitoxin, MazEF system), *repA* (plasmid replication initiation protein A), *klcA* (anti-restriction protein), *korC* (transcriptional repressor), *KPC-2* (β-lactamase KPC-2), Δ*TEM-40* (β-lactamase TEM-40 [93%]), IS*kpn27* (insertion sequence), *tn3* (Tn*3* family transposase), *DUF4158* (protein of unknown function), IS*APUA1* (insertion sequence of IS*4* family), *tnA* (Tn*3* transposase), *tniB* (Tn*5090*/Tn*402* recombination protein), IS*EC33* (insertion sequence), and *msrB* (methionine sulfoxide reductase B). Panel B was generated using Easyfig v2.2.5.

The *bla*_KPC-2_ carbapenemase gene was carried by the 51.2-kb IncP-6 plasmid (pErO89H7_5) along with two additional β-lactamase (*bla*_OXA-10_ and a truncated *bla*_TEM-40_) and macrolide [*mph(A)*], chloramphenicol (*cmlA1*), fluoroquinolone (*qnrVC4*), aminoglycoside [*ant(3″)-Ia*], and trimethoprim (*dfrA14*) resistance genes (Table 2). BLASTn analysis against the NCBI database revealed that pErO89H7_5 shares the highest similarity (62% coverage, 99.93% identity) with five plasmids reported in *Klebsiella oxytoca* KOX3 from China (KPC_PKOX3, KY913901), *Citrobacter freundii* 121SC21 from Spain (p121SC21-KPC2, NZ_LT992437.1), *Pseudomonas aeruginosa* 10265 from China (p10265-KPC, KU578314), *Enterobacter hormaechei* ECL189 from China (pECL189-1, CP047966.1), and *Klebsiella quasipneumoniae* WW14A from Argentina (pWW14A-KPC2, NZ_CP080103.1). It also showed 99.97% sequence identity with 42.0% coverage to pWW19C-KPC2 from *Enterobacter asburiae* WW19C (Argentina, NZ_CP080110.1) (Fig. 3A). pErO89H7_5 contains a unique ~20 kb region absent from previously described plasmids. A ~14 kb segment carries the *bla*_KPC-2_ gene within a Tn*3-*based transposon interrupted by an IS*Apu* element. Its core structure is ΔIS*Kpn6*/*bla*_KPC-2_/Δ*bla*_TEM-1_/IS*Kpn27*. The IS*Kpn6-bla*_KPC-2_-IS*Kpn27* cassette has been reported in diverse plasmids worldwide (44). Notably, the genetic environment surrounding *bla*_KPC-2_ remains highly conserved across different bacterial species. Typically, *bla*_KPC-2_ is embedded in the context IS*Kpn27*–Δ*bla*_TEM-40_–*bla*_KPC-2_–ΔIS*Kpn6–korC–klcA–ΔrepA* without mutations or rearrangements, as observed in the closely related IncP-6 plasmids pWW14A-KPC2 and p121SC21-KPC2, selected for comparison based on high sequence similarity (>95%) with our plasmid (Fig. 3B). Mating-out assays as previously described (3) demonstrated that the IncP-6 plasmid could be efficiently transferred at high frequency to *E. coli* J53 (2.0 × 10⁻^3^) and conferred an MDR phenotype (Table 1). Agarose gel analysis of the transferred plasmid confirmed a plasmid size of 51 kb (Fig. 2). Although the plasmid carries the mobCDE and oriT mobilization elements, mandatory for transfer initiation, the absence of T4CP and T4SS genes limits its ability for autonomous conjugation (45). Globally, the *bla*_KPC-2_ gene has been reported on many broad-host-range plasmids, including IncP (46). Plasmid pErO89H7_5 also carried genes conferring resistance to heavy metals (e.g., mercury) and disinfectants (e.g., quaternary ammonium compounds).

The IncM1 plasmid (pErO89H7_2) of 118.5 kb harbored two additional β-lactamase genes conferring resistance to expanded-spectrum cephalosporins (*bla*_SHV-12_ and *bla*_LAP-2_), as well as a fluoroquinolone resistance gene (*qnrS1*) (Table 2). Notably, *bla*_SHV-12_ and *qnrS1* are more commonly associated with IncX3 plasmids (47). The IncFIB/IncFII plasmid (pErO89H7_3) of 114.7 kb lacked AMR genes but encoded heavy metal resistance for copper (*pco*, *cop*, and *cus*), silver (*sil* genes), and tellurium (*ter* genes) as well as heat/stress survival proteins, and plasmid maintenance genes (*repA, repB*, *parM*, and *flmA*) with conjugation-associated genes (*traL*, *traA*, *traK*, and *traI*), suggesting a conjugative plasmid with potential for horizontal transfer and stabilization via toxin–antitoxin systems (*hok*). Similarly, the five additional plasmids of 85, 40, 5, 2.5, and 2.4 kb did not carry any resistance genes.

Chromosomal resistance determinants included *bla*_MIR-3,_ the aminoglycoside resistance genes *aac(6′)-Ib* and *aadA1*, and the *OqxA/OqxB* efflux pumps conferring reduced susceptibility to chloramphenicol, ciprofloxacin, nalidixic acid, and trimethoprim. Fluoroquinolone resistance-associated mutations were detected in *gyrA* (S83L), *parC* (S129A and S57T), and *parE* (I355T) (Table 1). The observed tetracycline resistance may be linked to a frameshifted TetR-family transcriptional regulator affecting efflux pump regulation (Table 1, Fig. 3A). Additionally, multiple mutations in phoP, phoQ, mgrB, pmrA, and a premature stop codon in pmrB likely account for the high-level colistin resistance (MIC > 8 mg/L) observed in Er O89H7 (Tables 1 and 2) (24).

## DISCUSSION

To the best of our knowledge, this study provides an in-depth genomic characterization of the first characterized and published clinical KPC-2-producing *Enterobacterales* from Lebanon. It is likely that KPC-producing organisms are underreported in Lebanon, potentially due to lack of reliable diagnostic tests, leading to an underestimation of their prevalence and clinical impact. Notably, this work represents the first identification of *Er* belonging to ST422 and of a highly conjugative IncP-6 plasmid carrying the *bla*_KPC-2_ gene in Lebanon, highlighting its potential role in KPC-2 dissemination. Carbapenemase screening in Lebanon remains limited, mostly relying on phenotypic tests, with few centers using molecular assays for rapid detection of KPC, NDM, VIM, or OXA-48-like gene (3). As similar IncP-6 plasmids have been detected in diverse bacterial species from clinical, environmental, water, and wastewater sources across geographically distant regions, it highlights the risk of horizontal transfer of *bla*_KPC-2_ gene between these compartments. Alarmingly, *Er* O89H7 was resistant to carbapenems and colistin, two antibiotics used as last-resort antimicrobials in Lebanon. Our results highlight an urgent need for improved carbapenemase screening and detection in clinical laboratories and for enhanced genomic surveillance of MDR bacteria to implement intervention strategies to control their spread in Lebanon and beyond.

## Data Availability

The assembled genomes of *E. roggenkampii* O89H7 and its transconjugant *E. coli* O123D5 have been deposited in the GenBank database under BioProject number PRJNA1262734 and accession number JBNYWS000000000.
